# Over the rainbow

**DOI:** 10.1186/s12915-015-0171-z

**Published:** 2015-08-06

**Authors:** Emma Saxon

**Affiliations:** BMC Biology, BioMed Central, 236 Gray’s Inn Road, London, WC1X 8HB UK

## Abstract

Shear stress in arteries, which is a measure of the force exerted by blood flow on the arterial wall, is associated with the location of lipid plaques that cause heart disease. In this study, a mathematical model of shear stress was combined with cross-sectional x-ray images of an artery taken using Computed Tomography (CT) scanning, allowing the authors to explore patterns of shearing stress and shed light on the role of arterial architecture in heart disease.

## Commentary

**Fig. 1.**
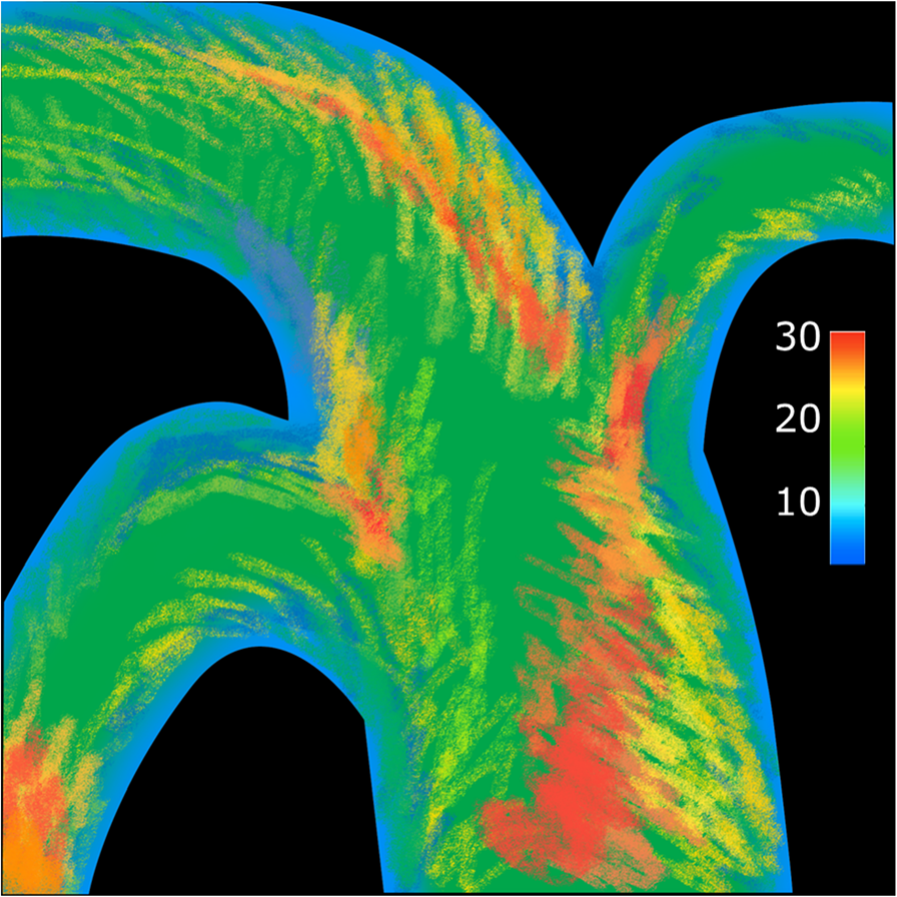
An example of shear stress patterns in an artery. Shear stress is measured in dyne/cm^2^, represented on the image by a rainbow scale from dark blue (0) to red (30)

Rainbow colour scales, which usually run from dark blue (low values) to red (high values), are commonly used in several different types of biological images: functional magnetic resonance imaging (fMRI) scans showing activity in the brain, gene expression data in microarrays, or in this case, the levels of shear stress in arteries, expressed as unit of force/unit of area (in this case, dyne/cm^2^). One problem with the rainbow scale is that it can make relatively small differences seem large: the red sections of Fig. 1 appear very different from the yellow. However, the colour scale shows that the difference between yellow and red is actually much smaller than it appears visually, in the context of the whole colour scale range.

The opposite can also be true. In Fig. 1, green spans just under half of the whole colour scale, and differences between values at the lower and higher ends of the middle range are somewhat masked. These differences could be better represented with a diverging scale, running between two colours only. As long as these colours are not red and green, a diverging scale would also be more easy to interpret for red-green colour blind individuals. Michelle Borkin and co-workers at Harvard University recently found that a diverging scale increased accuracy of interpretation relative to the rainbow scale in the case of arterial shearing images [1], challenging the legitimacy of using rainbow scales in this field.
